# Role of Ion Size and Hydration in Competitive Adsorption of Alkaline Earth Metals on TiO_2_ Nanoparticles: Experimental and Molecular Dynamics Insights

**DOI:** 10.1021/acsomega.5c06067

**Published:** 2025-12-15

**Authors:** Tilen Berglez, Boštjan Genorio, Goran Dražić, Jurij Reščič, Klemen Bohinc

**Affiliations:** † Faculty of Chemistry and Chemical Technology, 112794 University of Ljubljana, Večna pot 113, 1000 Ljubljana, Slovenia; ‡ Department of Materials Chemistry, National Institute of Chemistry, Hajdrihova ulica 19, 1000 Ljubljana, Slovenia; § Faculty of Health Sciences, 112794 University of Ljubljana, Zdravstvena 5, 1000 Ljubljana, Slovenia

## Abstract

Interactions between alkaline earth metal cations (M^2+^) and titanium dioxide nanoparticles (TiNPs) critically influence the TiNP surface charge and colloidal stability in biological and environmental systems. Here, we systematically investigate how the cation size, hydration, and concentration affect the interfacial adsorption of Mg^2+^, Ca^2+^, Sr^2+^, and Ba^2+^ on TiNPs. X-ray photoelectron spectroscopy and transmission electron microscopy provide direct evidence of cation adsorption on the TiNP surface and reveal ion-specific differences in binding extent and distribution. Zeta potential measurements across a broad pH and concentration range further demonstrate that adsorption increases with cation molar mass and leads to pronounced overcharging of the TiNP surface—an effect that is strongly ion-specific and concentration-dependent. To interpret these experimental findings at the molecular level, molecular dynamics simulations were used to identify binding sites, compute potential of mean force (PMF) profiles, and estimate adsorption free energies, providing insight into ion-specific cation–surface interactions. We find that elevated concentrations of background monovalent salt significantly reduce the level of M^2+^ adsorption, highlighting the importance of competitive adsorption at the nanoparticle–water interface. These results elucidate key factors governing TiNP surface chemistry in complex aqueous environments and inform the design of nanomaterials for biological and ecological applications.

## Graphical abstract

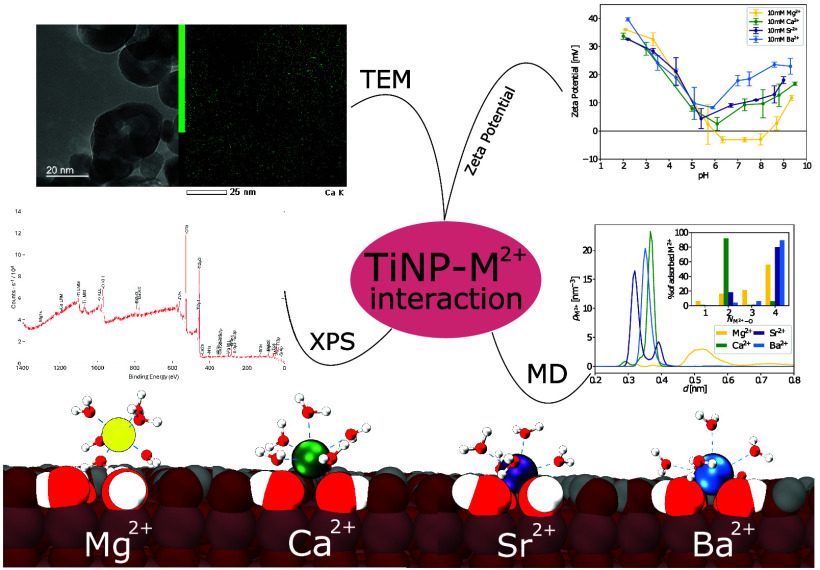

## 1 Introduction

Titanium dioxide nanoparticles (TiNPs), which are among the most widely studied and used metal oxide nanoparticles, have numerous applications in industry and medicine, such as in drug delivery systems, titanium implant coatings, and as adsorbents for wastewater remediation.^1–5^ In aqueous suspensions, TiNPs acquire a pH-dependent surface charge and readily adsorb various organic and inorganic species, which often change their surface charge.^6,7^ Adsorbed polyelectrolytes and ions can also be used to intentionally alter the surface properties of the nanoparticles and fine-tune the behavior of TiNPs for different applications.^8^

In this study, a subset of alkaline earth metal ions (Mg^2+^, Ca^2+^, Sr^2+^, and Ba^2+^) was investigated for their interaction with TiNPs by using experimental and computational methods. For clarity, these ions are collectively termed M^2+^ throughout this work. This selection of ions allows for the study of the effects of the size of the divalent ion and its hydration on the interaction with TiNPs. The advantage of using these ions is also their good solubility and low hydroxide formation below pH 10,^9^ which allows the study of the effect of pH on the adsorption of divalent ions to TiNPs.

The interaction of TiNPs with Ca^2+^ and Mg^2+^ ions is important, as these ions are among the most abundant divalent metal ions in biological and ecological systems. For example, understanding the effects of Mg^2+^ and Ca^2+^ on the properties of TiNP suspensions is critical for biomedical applications of TiNPs, as these ions can greatly modify the properties of TiNPs, especially their surface charge, which affects their ability to adsorb and release other molecules and ions, as well as the aggregation of nanoparticles in aqueous media. In drug delivery systems, this can affect the release of compounds depending on the concentration of Ca^2+^ and Mg^2+^.^10^ If TiNPs are used as adsorbents for the removal of organic or inorganic substances, Mg^2+^ and Ca^2+^ can also influence the efficiency of their removal.^11^ This is important for the removal of toxic metal or organic ions from wastewater, where Ca^2+^ and Mg^2+^ can compete for adsorption with other charged species. Sr^2+^ and Ba^2+^ ions are less biologically relevant but can be ecologically problematic due to their toxicity. 90-Sr^2+^ is a hazardous pollutant that can be released into the environment by nuclear power plants and can harm living organisms due to its radioactivity.^12^ In addition, due to its similarity to Ca^2+^, Sr^2+^ can replace Ca^2+^ in bones, further increasing its potential carcinogenicity.^13^ Ba^2+^ is also highly toxic to humans and can cause changes in heart rhythm and paralysis.^14^ Therefore, the interaction of TiNPs with Sr^2+^ and Ba^2+^ is particularly relevant in relation to the removal of ions from wastewater using TiNPs as adsorbents.^15,16^

The adsorption of charged species on TiNPs can be investigated using a variety of methods.^17–19^ Transmission electron microscopy (TEM) and X-ray photoelectron spectroscopy (XPS) are suitable methods for studying the adsorption of metal ions on TiNPs. TEM can be used for TiNP characterization and to determine the concentration of adsorbed cations on the surface of the TiNPs.^20^ XPS can also be used to obtain an estimate of the amount of adsorbed ions.^21^ Traditionally, the adsorption of metal ions on TiNPs in suspension is investigated indirectly by complexometric titration of the metal ions left in suspension after the removal of TiNPs with adsorbed ions.^11^ XPS and TEM present an interesting alternative, as the adsorbed ions can be determined directly on the surface of TiNPs, while TEM also allows the distribution of the ions on the surface to be investigated. Zeta potential measurements are routinely used to study the charge properties of TiNPs with adsorbed species.^22^ To obtain a more complete picture of the adsorption process at the atomic level, molecular dynamics computer simulations (MD simulations) are often the method of choice for modeling the adsorption of charged species on metal oxide surfaces.^23–25^

The interaction of M^2+^ with TiNPs in aqueous suspensions has already been studied experimentally;^26–29^ however, there still exist contradicting claims when it comes to the affinity trends of these ions for the TiNP surface. Malati and Smith have found that for both rutile and anatase forms of TiNPs, the adsorption affinity of M^2+^ increases in the following order: Ba^2+^ < Sr^2+^ < Ca^2+^.^28^ On the other hand, the exact opposite order for adsorption on rutile NPs was proposed by Jang and Fuerstenau (Mg^2+^ < Ca^2+^ < Sr^2+^ < Ba^2+^), based on adsorption and electrokinetic measurements,^29^ which were limited to relatively low ion concentrations (up to 1 mM). Since then, not much attention has been paid to these contradictory findings. These discrepancies may arise from impurities in nanomaterials used and reported by Malati and Smith, where the anatase samples had significant amounts of SO_3_ present, which could be present in suspension as another charged species adsorbing onto the surface. Our results show that the presence of multiple charged species strongly affects their individual adsorption affinities on nanoparticles. An additional reason could be the difference in the background electrolyte concentration: Malati and Smith used 100 mM KNO_3_, where K^+^ could compete with M^2+^ for adsorption, while Jang et al. introduced monovalent ions only through pH adjustment with KOH.

In this work, we address these inconsistencies to fill the gap in understanding the adsorption of M^2+^ on anatase TiNPs by combining experimental measurements with extensive computer simulations. We selected the anatase form of TiNPs because Malati and Smith reported the same M^2+^ affinity order for both rutile and anatase, whereas Jang and Fuerstenau studied only rutile and found the opposite order, suggesting that anatase had not been thoroughly examined in this context. Using TEM and XPS, we determined how the affinity of M^2+^ to adsorb on TiNP changes with the molar mass and size of the cation. Zeta potential measurements were performed to estimate the effects of M^2+^ on the charge properties of TiNPs in suspension for a wide concentration range of cations (0.1–100 mM). Molecular dynamics simulations of M^2+^ solutions in contact with the TiNP surface were performed to determine the binding modes of M^2+^ on the TiNP surface, the profiles of the mean force (PMF) of M^2+^ during adsorption, and the effect of M^2+^ adsorption on the surface charge of the TiNP. As far as we know, up to this point, there was no systematic study in the literature on the adsorption of M^2+^ on TiNPs using MD simulations. Lastly, the effect of the addition of monovalent ions (in this study Na^+^) on the adsorption of M^2+^ was investigated using experimental techniques XPS and MD simulations.

## 2 Materials and Methods

### 2.1 Materials

Spherical TiO_2_ nanoparticles were purchased from Merck (product number 637262) and initially characterized by XRD (Figure S1), TEM (Figure S2), and BET (see the Supporting Information). The primary particle size of the nanoparticles was about 25 nm, and the crystal structure was that of anatase. To prepare the solutions of divalent metal ions, MgCl_2_·6H_2_O, CaCl_2_·2H_2_O, SrCl_2_·6H_2_O, and BaCl_2_·2H_2_O were dissolved in MQ water to obtain stock solutions of metal ions (*c* = 0.2 M). 0.1 M HCl and 0.1 M NaOH were used for pH adjustment. A stock solution of TiNPs in water (1 g/L) was prepared and sonicated for 15 min using an ultrasonic probe (Hielscher UP100H, 100 W probe) to achieve dispersion of the TiNP powder. MQ water was used for the preparation of all of the suspensions and solutions.

### 2.2 Zeta Potential Measurements

For the zeta potential measurements, the TiNP stock solution and the metal ion stock solutions were mixed and then diluted to 20 mL with MQ water so that the final TiNP concentration was 0.1 g/L and the metal ion concentration was as required (0.1–100 mM). The pH of the samples was then adjusted by using a few drops of 0.1 M HCl or 0.1 M NaOH. The samples were then left to equilibrate overnight. Before measurement, each sample was sonicated again briefly (1 min), and its pH was measured. This was done because the addition of metal ions significantly reduces the stability of the suspension even at low concentrations, and the short sonication ensured that the sample was stable over the entire duration of the measurement without heating the sample considerably. The zeta potential was then measured using a LiteSizer 500 from Anton Paar. The sample was first equilibrated in a measuring vessel for 2 min to ensure that the measurement temperature was 25 °C. For each sample, the measurement of 200 runs was repeated four times, and the results were averaged.

### 2.3 Determination of Metal Ion Adsorption Using XPS and TEM

Using XPS and TEM, which allow the determination of the molar proportions of the elements in a dry sample, it is possible to directly determine the ratio between the number of Ti atoms in the sample and the number of metal ions in the sample adsorbed on the surface. In this way, several different adsorbed metal ions can also be determined simultaneously with just one measurement.

The sample preparation was very similar to the zeta potential measurements, with the only difference that after equilibrating the samples overnight, a final pH measurement was performed, and the pH was readjusted, if necessary, to reach the desired pH (within 0.1 units of pH). Samples were then allowed to equilibrate again for 3 h before being centrifuged at 4300 rpm and washed with a NaOH solution with a pH value equal to the pH of the samples (usually pH 9). After washing, the samples were dried in an oven at 60 °C overnight or until completely dry.

The samples were analyzed using a probe-Cs-corrected high-resolution scanning transmission electron microscope (HR-STEM) (JEOL ARM 200 CF, Tokyo, Japan) operated at an accelerating voltage of 80 kV. Elemental mapping was performed using an energy-dispersive X-ray spectroscopy (EDS) detector (JEOL Centurio 100) coupled to the microscope. SPI 300 mesh lacey-carbon-coated Cu grids were used as a support for TiNP. The dried TiNP powder was transferred directly onto the grids, avoiding the commonly used ultrasonic dispersion in alcohol so as not to desorb the ions. The area investigated was approximately 100 nm × 100 nm.

XPS measurements were conducted by using a Versa Probe 3 AD (PHI, Chanhassen, USA) equipped with a monochromatic Al Kα X-ray source. The source operated at an accelerating voltage of 15 kV and 46 W. Powder samples were mounted on double-sided Scotch tape and positioned at the center of the XPS holder. Spectra were acquired for each sample over a 200 μm × 200 μm analysis area with a charge neutralizer activated during data collection. Survey spectra were measured using a pass energy of 224 eV and a step size of 0.2 eV. High-resolution (HR) spectra were recorded with a pass energy of 27 eV and a step size of 0.05 eV. To ensure high-quality spectral data with a good signal-to-noise ratio, at least 10 sweeps were performed for each measurement. The energy scale of the XPS spectra and any possible charging effects were corrected by referencing the C=C peak in the C 1s spectrum with a binding energy of 284.8 eV. Spectral quantification was carried out using MultiPak 9.9.1 software with Shirley background correction (examples given in the Supporting Information in Figure S5).

### 2.4 Molecular Dynamics Computer Simulations

#### 2.4.1 TiO_2_ Model

The parameters of the TiO_2_ model and the initial configuration of the TiO_2_ slab used for the simulations were taken from the work of Biriukov and colleagues.^30^ This model was parametrized for use with the SPC/E water model, which was also used in this work. To approximate different conditions in terms of pH, slabs of TiO_2_ with different surface charge densities were prepared. At low pH, where TiO_2_ is positively charged, a slab with σ = +0.1 C/m^2^ was prepared. To approximate the conditions around the IEP of the nanoparticles, a slab with σ = 0 C/m^2^ was used, and for high pH values well above the IEP, a slab with σ = −0.1 C/m^2^ was used. Each surface with a specific σ was prepared in two variants: a hydroxylated (H-TiNP) and nonhydroxylated (NH-TiNP) surface (Figure S3 in the Supporting Information). These two variants represent two possible representations of the interface between TiO_2_ and water and were also used in the original work by Předota and colleagues.^31^ The H-TiNP surface (Figure S3a in the Supporting Information) represents a case where the surface is covered with hydroxyl groups due to the dissociation of water molecules, while the NH-TiNP model (Figure S3b) represents a state where the surface is covered with adsorbed water molecules (which are adsorbed during the MD simulation) strongly bound to the Ti atoms of the surface.^32^ In the case of the NH-TiNP surface, a smaller number of hydroxyl groups is added for the negatively charged surface to obtain the desired σ. The size of a simulation box was the same in all simulations, measuring 7.796 × 7.080 × 15 nm, with the TiNP slab (around 2 nm thick), centered in the box with the surface perpendicular to the *z*-axis.

In the original parametrization of this model, the ECCR method (electronic continuum correction with rescaling) was used to model charged surfaces and ions in solution. This was due to the overestimation of the interatomic interaction that occurs when using fully charged models.^33^ To obtain a more realistic modeling of the charged species, their partial charges are scaled by the inverse square root of the electronic part of the dielectric constant of the solvent, which in water is around 0.75.^34^ We scaled partial charges of charged nanoparticle surfaces, as well as those of metal and chloride ions that were part of the simulations with charged surfaces.

#### 2.4.2 Simulation Details

All MD simulations were performed using the GROMACS software package.^35^ The SPC/E water model was used to model water molecules.^36^ All divalent metal ions were modeled using the models developed by Li and colleagues, which were specifically parametrized for use with the SPC/E water model.^37^ Divalent ions modeled using these parameters have been used in a variety of systems, with biological molecules, crystalline materials, and charged surfaces.^38–40^ However, it is important to note that reproduction of divalent ion solution behavior using classical force fields can be problematic in comparison to monovalent ion modeling.^41^ This is due to the larger charge of divalent ions, which can lead to overbinding of water molecules and inaccurate modeling of cation–anion interactions compared to real solutions.^42^ The most problematic of all the chosen divalent cations is Mg^2+^, for which it was shown that the standard combination rules cannot be used to achieve the experimental activity coefficient at high ion concentrations.^43^ An approach that can be used to mitigate these errors is the scaling of ion charges, which was applied in this work for all charged groups (using the ECCR method).^44^ Another approach to determine the adsorption energy between divalent ions and TiO_2_ or the binding sites of divalent ions on TiO_2_ would be with the use of *ab initio* MD simulations.

The leap-frog algorithm was used to integrate Newton’s equations of motion, with an integration time step of 2 fs. For both van der Waals and Coulomb interactions, a cutoff distance of 1.4 nm was applied. The particle mesh Ewald method (PME) with a Fourier spacing of 0.12 nm was used to treat electrostatics.^45^ During the simulations, bonds involving hydrogen atoms were constrained using the LINCS algorithm.^46^ All simulations were performed at 298.15 K using a V-rescale thermostat (τ = 1 ps).^47^ Simulations in the *NPT* ensemble were carried out at *p* = 1 bar using a Berendsen barostat (τ = 5 ps).^48^ For the simulations involving the TiO_2_ slab, a semi-isotropic pressure coupling was applied, with the compressibility in the *xy*-plane equal to 5 × 10^–7^ bar^–1^ to avoid deformation of the crystal structure and errors in bonding across cell boundaries. For this reason, the simulations had to be run with the switch ‘periodic molecules’ set to ‘yes’. The compressibility in the *z*-dimension was set to 5 × 10^–5^ bar^–1^. Periodic boundary conditions in three dimensions were applied to all simulations.

First, energy minimization was performed using the steepest gradient descent method, followed by short *NVT* and *NPT* ensemble simulations (lasting 100 ps and 1 ns, respectively). Finally, 100 ns long *NPT* production run simulations were performed. The simulation trajectories were analyzed using the built-in GROMACS tools (pairdist and density functions). Further analyses were carried out using in-house Python scripts. The trajectories were visualized, and image rendering was done using VMD software.^49^

Umbrella sampling simulations were performed to calculate the potential mean force (PMF) profiles of M^2+^ during adsorption on the surface of H-TiNP. The last frames of the 100 ns production simulations were used as starting configurations for the steered MD simulations (lasting 1 ns), in which the selected adsorbed ion was pulled from the surface of the TiNP using a harmonic potential with a force constant of 10,000 kJ mol^–1^ nm^–2^ in the direction perpendicular to the surface of the TiNP. These simulations were used to obtain initial configurations (with a spacing of around 0.05 nm) for the umbrella sampling simulations. For each window, a 5 ns simulation was performed, in which the ion was held at a fixed distance from the TiNP surface using a harmonic potential with a spring constant between 1000 and 10,000 kJ mol^–1^ nm^–2^. PMFs were then calculated using the weighted histogram analysis method (WHAM), which is included in the GROMACS software package as the wham module.^50,51^

## 3 Results and Discussion

### 3.1 Adsorption Capacity of M^2+^ on TiNPs Determined Using TEM and XPS

The molar proportions of the elements in the dry sample were determined by TEM and XPS (Figure 1). To estimate the amount of adsorbed ions on the surface of the TiNPs, the molar fractions of adsorbed M^2+^ in the sample were divided by the molar fraction of titanium atoms in the sample. A higher value of *x*_M^2+^_/*x*_Ti_ indicates a larger amount of adsorbed M^2+^ on the TiNPs. The values obtained by TEM and XPS cannot be directly compared because XPS mainly examines the surface of the material to a maximum depth of about 5–10 nm and provides information about the average composition on a relatively large surface area. However, the TEM electron beam is transmitted through the sample, resulting in a higher molar fraction of Ti compared to the XPS measurements, which leads to lower ratios of *x*_M^2+^_/*x*_Ti_ in the TEM measurements compared to XPS measurements.

**Figure 1:**
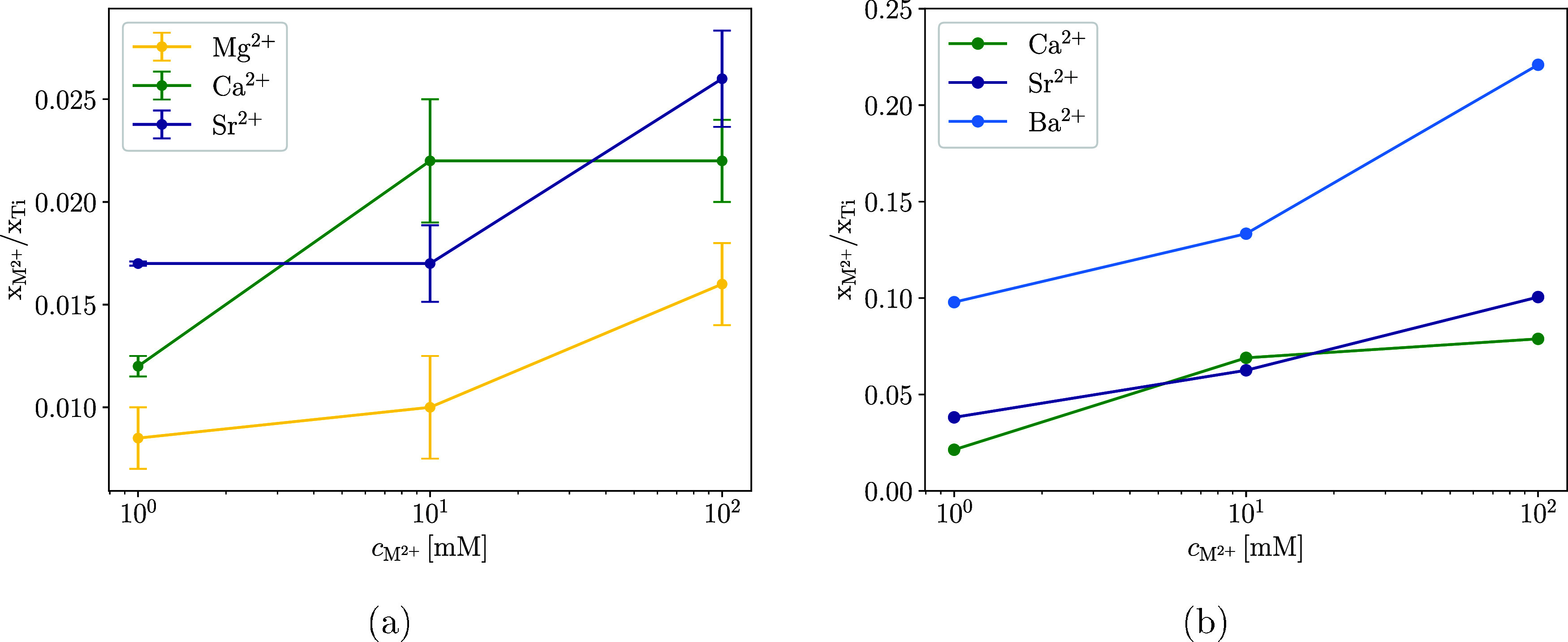
*x*_M^2+^_/*x*_Ti_ for different concentrations of M^2+^ (*c*_M_^2+^) in TiNP suspensions, determined by TEM (a) and XPS (b), and pH of the suspensions before centrifugation was adjusted to 9. The TiNP concentration in the suspension before centrifugation and drying was 0.2 g/L in all samples. The error in determination of *x*_M^2+^_/*x*_Ti_ with XPS did not exceed 10%.

Both XPS and TEM were used to determine the adsorption of metal ions as not all M^2+^ on TiNP could be accurately quantified using a single technique due to experimental difficulties. With TEM, the adsorption of Ba^2+^ on TiNPs could not be reliably measured because the Ti and Ba peaks overlapped in the EDS spectra, and the quantification of Ba could not be done dependably. In XPS measurements, similar problems occurred with peak overlap in samples with adsorbed Mg^2+^, where the Ti 3s peak overlapped with the Mg 2p peak. In addition, Mg 1s peak quantification did not produce consistent or interpretable results. Therefore, to obtain a complete picture of the affinity of M^2+^ for adsorption on TiNPs, Figure 1a,b must be considered together. Both graphs show that the amount of adsorbed ions increases with increasing molar mass of the cation. Ba^2+^ adsorbs in significantly larger quantities than Ca^2+^ and Sr^2+^, which adsorb in a similar amount. Adsorption of Mg^2+^ is significantly lower than that for the other cations. This is consistent with the work of Jang et al., where the adsorption of M^2+^ was measured for rutile nanoparticles with unknown particle geometry and size.^29^ It is important to note that well-defined anatase TiNPs were used in this work (see the Supporting Information for more details), which shows that the crystal structure of TiNPs does not seem to affect the order of interaction strength of M^2+^ with TiNPs.

Figure 2 shows the elemental mapping of adsorbed ions Mg^2+^ and Ca^2+^ to TNPs. On a similar size scale, it can be seen that the density of adsorbed Ca^2+^ (green dots in Figure 2f) is much higher than the density of adsorbed Mg^2+^ (green dots in Figure 2c). Both types of cations are quite evenly positioned on the surface of the TiNPs.

**Figure 2:**
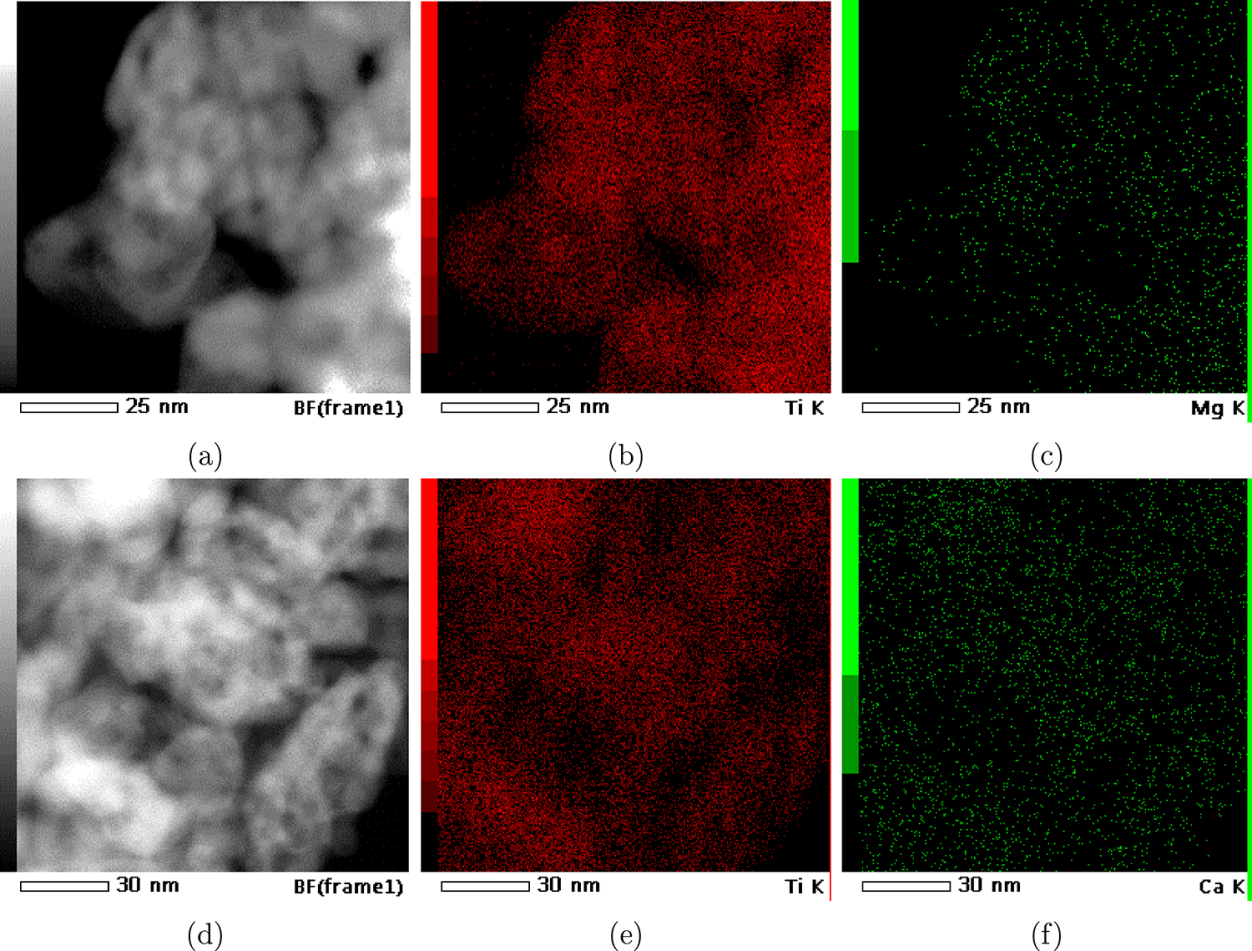
TEM elemental mapping of TiNPs with adsorbed Mg^2+^ ((a) TEM micrograph, (b) mapping of Ti, and (c) mapping of Mg) and with adsorbed Ca^2+^ ions ((d) TEM micrograph, (e) mapping of Ti, and (f) mapping of Ca).

Using XPS, it is possible to detect and quantify multiple ion species within the same sample. To evaluate competitive adsorption, a TiNP sample was prepared from a suspension containing all investigated M^2+^ cations at a concentration of *c*_M_^2+^ = 10 mM. The value of *x*_M^2+^_/*x*_Ti_ was determined for each cation using XPS, and the results are presented in Table 1. In all cases, *x*_M^2+^_/*x*_Ti_ values were lower than those observed in samples where only a single cation was present (Figure 1b). Despite the overall reduction, the relative order of cation affinity for the TiNP surface remained consistent with the notable exception of Sr^2+^, which showed significantly greater adsorption than Ca^2+^. This suggests that when cations compete directly for adsorption sites, differences in surface affinity are magnified. The *x*_M^2+^_/*x*_Ti_ ratio for Mg^2+^ is included but should again be interpreted with caution, although the near absence of the Mg 1s signal indicates negligible adsorption of Mg^2+^ on TiNP (see Figure S4 in the Supporting Information).

**Table 1:** *x*_M^2+^_/*x*_Ti_ for Cations in a Single Sample of TiNPs, Which Was Prepared from a Suspension That Contained All of the Divalent Ions at a Concentration of 10 mM at pH 9^a^

	*x*_M^2+^_/*x*_Ti_
Mg^2+^	<0.005
Ca^2+^	0.010
Sr^2+^	0.021
Ba^2+^	0.036

a The concentration of TiNPs was equal to 0.2 g/L.

As mentioned above, Malati and Smith determined an opposite order of affinity of cations for adsorption: Ca^2+^ > Sr^2+^ > Ba^2+^.^28^ Their measurements were performed in suspensions containing 100 mM KNO_3_, where *K* ^+^ could compete with divalent ions for adsorption. To determine whether the monovalent salt could strongly affect divalent cation adsorption on TiNPs, further XPS experiments were performed.

With increasing concentrations of Na^+^ in the suspension of TiNPs with divalent ions, the amount of adsorbed divalent ions decreases (Figure 3). This means that the concentration of monovalent ions in suspension is an important parameter when it comes to studying the adsorption of divalent ions on the surface of TiNPs. The concentration of Na^+^ (*c*_Na^+^_) affects the adsorption of all considered ions similarly. The amount of adsorbed M^2+^ ions when *c*_Na_^+^ = 5 × *c*_M^2+^_ falls to around 66–78% of the amount of adsorbed divalent ions without added Na^+^. The addition of Na^+^ does therefore not affect the order of affinity of M^2+^ for the TiNP surface.

**Figure 3:**
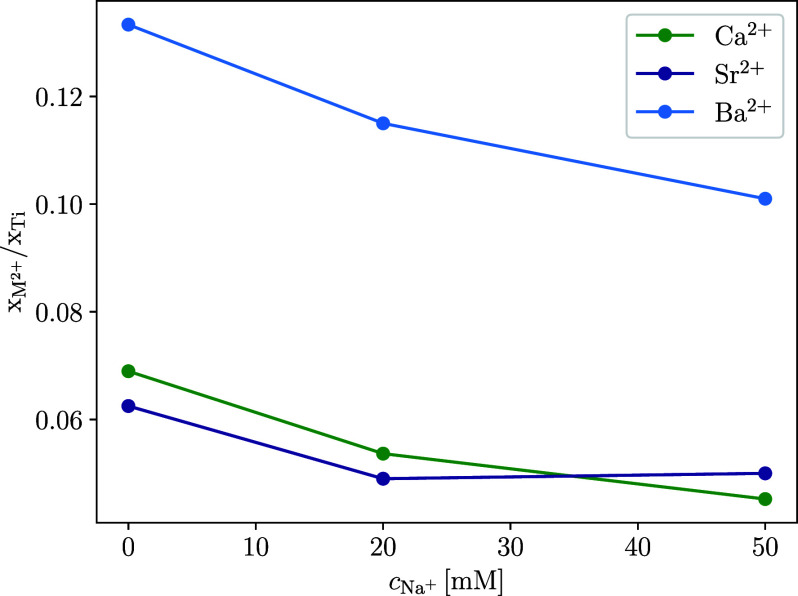
*x*_M^2+^_/*x*_Ti_ ratios for different concentrations of Na^+^ (*c*_Na^+^_) in TiNP suspensions, determined using XPS. The concentration of M^2+^ was 10 mM in all samples, while the concentration of TiNPs in the suspension before centrifugation and drying was 0.2 g/L.

### 3.2 Influence of M^2+^ on the Zeta Potential of TiNPs

Figure 4 shows the zeta potential of TiNPs as a function of the suspension’s pH for different concentrations of M^2+^. For pH values below the isoelectric point of the nanoparticles (pH_IEP_ = 5.5), where TiNPs are positively charged, the addition of cations does not have a pronounced or cation-specific effect. Differences between cations become apparent at pH values greater than pH_IEP_, where TiNP acquires a negative surface charge and adsorption becomes favorable. The adsorption of cations leads to an increase in the zeta potential, which is concentration- and cation-dependent. Even at a very low concentration of cations (*c*_M^2+^_ = 0.1 mM), there is an effect of different cation types on the zeta potential of TiNP (Figure 4a). The addition of Mg^2+^ leads to the smallest increase in the zeta potential (about 15 mV compared to bare TiNP), followed by Ca^2+^ and Sr^2+^ (an increase of around 18 mV compared to bare TiNP). Ba^2+^ ions cause the largest increase in zeta potential (around 25 mV compared to that of bare TiNP). This trend agrees well with adsorption measurements (Figure 1), previous literature data, and also confirms that the adsorption affinity on anatase TiNPs increases in order: Mg^2+^ < Ca^2+^ ≈ Sr^2+^ < Ba^2+^.

**Figure 4:**
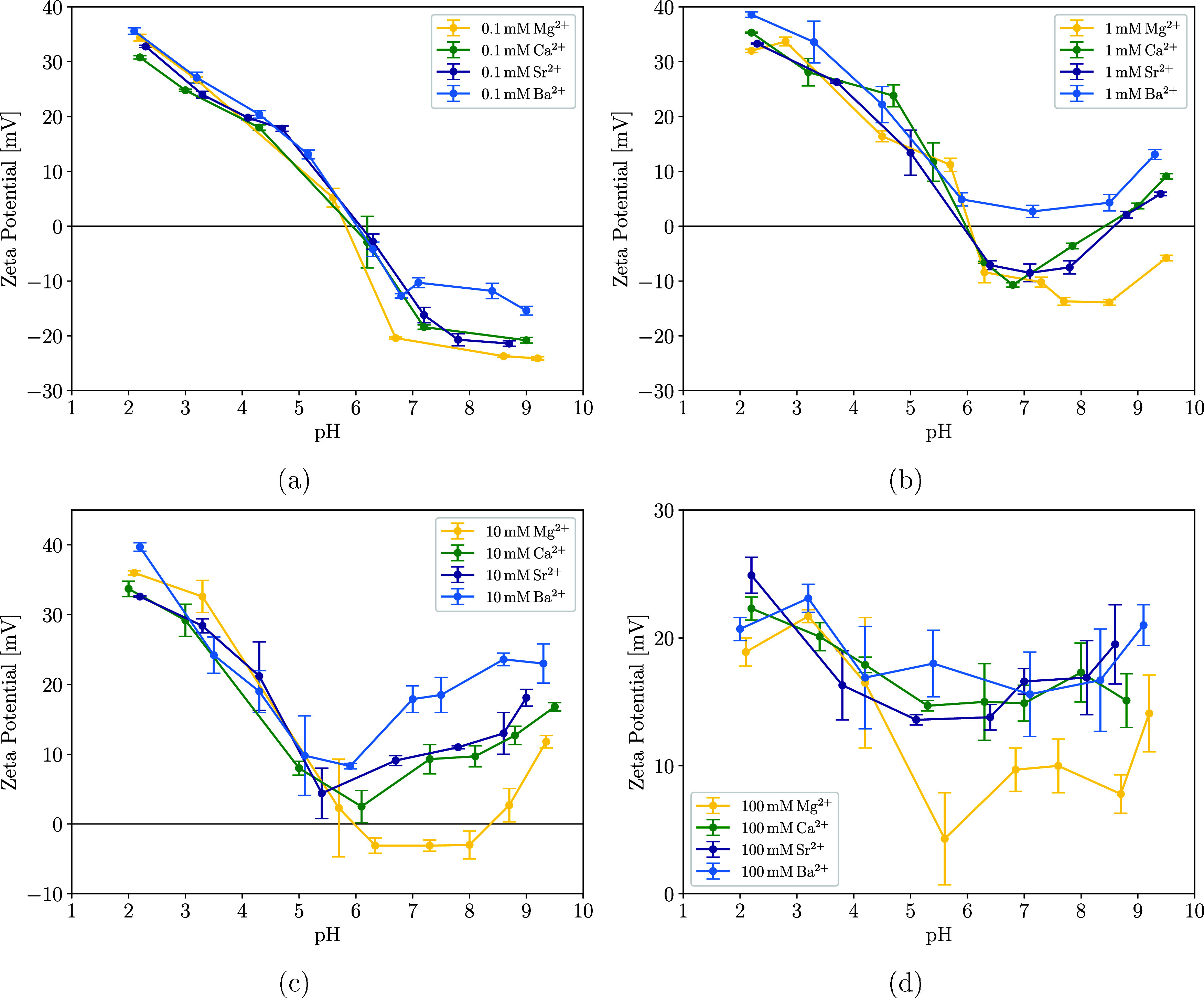
Dependence of the zeta potential on the pH value of the TiNP suspensions with added M^2+^ for a range of cation concentrations (0.1 mM (a), 1 mM (b), 10 mM (c), and 100 mM (d)). The TiNP concentration in the suspension for all measured samples was 0.1 g/L.

With increasing cation concentration, the zeta potential becomes less negative. When *c*_M^2+^_ increases to 1 mM, for Ca^2+^ and Sr^2+^, the zeta potential of the TiNPs becomes positive at pH above 8.5, which is known in the literature as the overcharging of nanoparticles.^52^ The adsorption of Ba^2+^ causes overcharging of the TiNPs in the whole pH range, with a minimum potential value of about 3 mV and a significant increase to almost 15 mV above pH 8.5. The most diverse zeta potential of TiNPs is achieved for *c*_M^2+^_ = 10 mM (Figure 4c) and 6 < pH < 8.5, where the zeta potential profiles of TiNPs with different ions are separated by about 5–10 mV, except for Ca^2+^ and Sr^2+^ that have a very similar influence on the zeta potential of TiNPs. Above pH 9, the zeta potential profiles of TiNPs with adsorbed ions start to converge. This effect may be the result of the formation of metal ion hydroxides at higher pH, which form particles due to their low solubility and have a positive zeta potential (approximately 20 mV).^53–55^

For *c*_M^2+^_ = 100 mM (Figure 4d), the zeta potential remained strongly positive throughout the pH range under consideration. This high ionic strength of the suspension reduces the stability of the suspension due to the lower electrostatic repulsion between nanoparticles,^56^ and consequently, the experimental errors are larger compared to the lower ionic strength. Therefore, it is almost impossible to evaluate the differences between zeta potentials for most ions at *c*_M^2+^_ = 100 mM. The comparison between TiNP suspensions with *c*_M^2+^_ = 10 and *c*_M^2+^_ = 100 mM shows that the maximum zeta potential does not change with the increase in concentration, although the amount of adsorbed ions continues to increase (as shown by XPS and TEM measurements). This plateau in maximum zeta potential beyond *c*_M^2+^_ = 10 mM is likely due to enhanced counterion (Cl^–^) condensation at higher ionic strengths, which effectively screens the surface charge and limits further overcharging of TiNPs. However, at *c*_M^2+^_ = 100 mM, an increase in zeta potential is observed within the pH range of 5–8.5, resulting in a relatively stable zeta potential across the full pH range. Only TiNPs with added Mg^2+^ showed a decrease in zeta potential around the isoelectric point of TiNPs, even at *c*_M^2+^_ = 100 mM, and their zeta potential remained lower than that of the other cations throughout the pH range considered.

### 3.3 Adsorption Modes of M^2+^ Adsorption on TiNP Surfaces Analyzed Using Molecular Dynamics Simulations

Figure 5 shows the number density profiles of the divalent metal ions (ρ_M^2+^_) perpendicular to the surface of the negatively charged TiNP slab for both TiNP surface models. Insets in Figure 5a,b are the % of adsorbed M^2+^ that are coordinated to a certain number of surface oxygen atoms (*N*_M^2+^–O_) of the corresponding TiNP surface.

**Figure 5:**
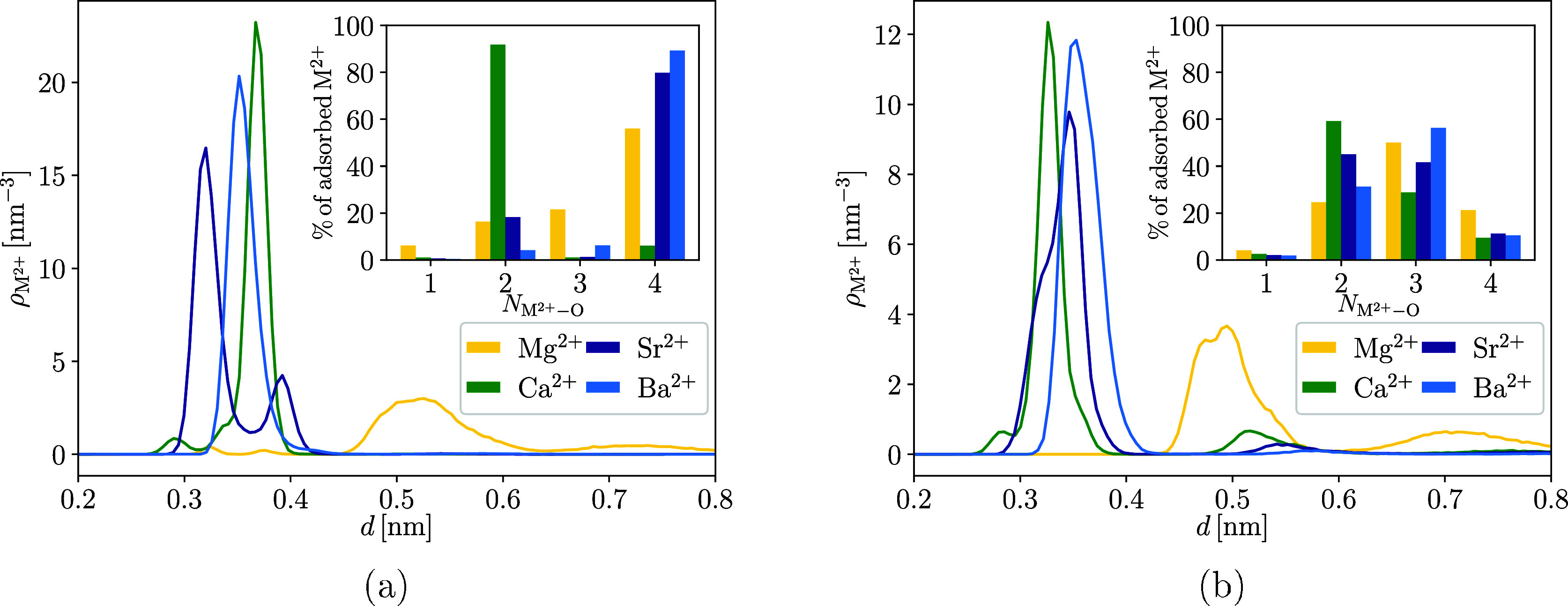
Number density of M^2+^ (ρ_M^2+^_) above the negatively charged H-TiNP (a) and negatively charged NH-TiNP (b) slab. The last row of the crystal lattice of the TiNP surface is set to *d* = 0 nm, which allows a comparison between the H-TiNP and NH-TiNP model. *c*(M^2+^) was about 200 mM in all simulations, which corresponded to 100 divalent cations in the simulation box. Insets in parts (a) and (b) are the figures representing the % of adsorbed M^2+^ that are coordinated to a certain number of surface oxygen atoms (*N*_M^2+^–O_) of the corresponding TiNP surface.

The ρ_M^2+^_ profiles for the negatively charged H-TiNP surface (Figure 5a) show that Mg^2+^ behaves quite differently from the other M^2+^ and adsorbs at a much greater distance from the surface (*d* ≈ 0.52 nm), although it has the smallest ionic radius. This is a consequence of the stronger hydration of Mg^2+^, which means that Mg^2+^ adsorbs fully hydrated.^57^ Mg^2+^ also has a low secondary peak (*d* ≈ 0.73 nm) in the density profile, which corresponds to a slightly increased concentration of Mg^2+^ next to the bilayer of cations and anions closest to the TiNP surface. The strong hydration of Mg^2+^ compared to other divalent cations is also evident in Figure 6, which shows the radial distribution functions (*g*(*r*)) between M^2+^ and oxygen atoms of water molecules calculated from MD simulations.

**Figure 6:**
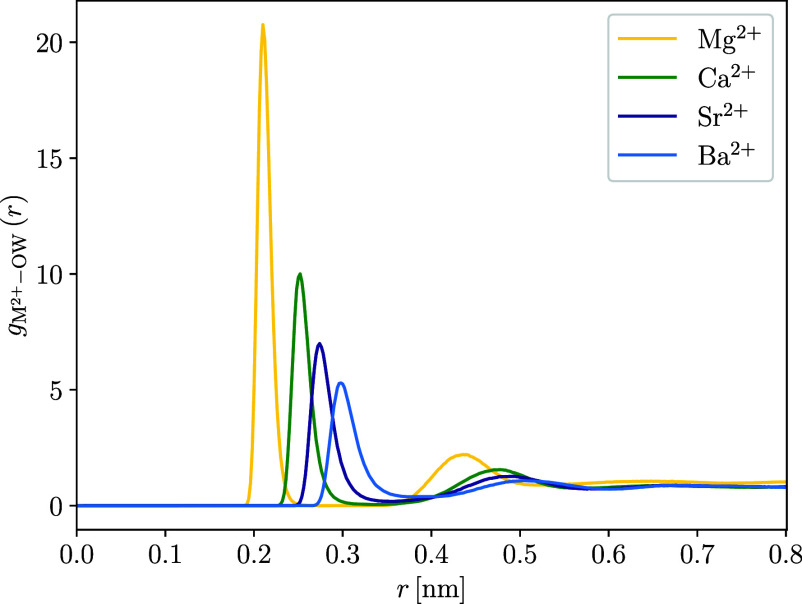
Radial distribution functions (*g*(*r*)) between divalent cations and oxygen atoms of water molecules calculated for the last 20 ns of the MD simulations with the TiNP surface. RDFs were calculated with inclusion of all divalent ions in the simulation box, regardless of whether they were adsorbed or not.

Next in the group of M^2+^ is Ca^2+^, which already shows a clearly different behavior from Mg^2+^. The first peak in ρ_Ca^2+^_ at *d* ≈ 0.29 nm belongs to the ions coordinated to four oxygen atoms of the TiNP surface, which are usually referred to as being adsorbed at the tetradentate sites (4D) on the TiNP surface.^58^ The first peaks of the density profiles of Sr^2+^ (*d* ≈ 0.32 nm) and Ba^2+^ (*d* ≈ 0.35 nm) also belong to the ions adsorbed at the 4D sites. The position of the first peak depends on the size of the ion, with Ca^2+^ adsorbing closest to the surface, followed by Sr^2+^ and Ba^2+^.

The position of the first peak in ρ_M^2+^_ is not the best indication of the overall adsorption of cations, as Ca^2+^ strongly prefers to adsorb further away from the surface, as shown by the much higher second peak in the ρ_Ca^2+^_ profile at *d* ≈ 0.37 nm. This peak corresponds to Ca^2+^ adsorbed at the bidentate site (2D) coordinated with two oxygen atoms of the TiNP surface. Around 90% of Ca^2+^ adsorbs at the 2D site (inset of Figure 5a), while the remaining Ca^2+^ adsorbs mostly at the 4D site. Considering that the smaller ions are more strongly hydrated, it can be concluded that Ca^2+^ prefers the 2D site, as it can adsorb there without losing as many of its coordinated water molecules as at the 4D site, where it is surrounded by 4 oxygen atoms of the TiNP surface. The adsorption of Sr^2+^ follows a similar trend, with a second peak in the ρ_Sr^2+^_ profile at *d* ≈ 0.39 nm. The major difference between Sr^2+^ and Ca^2+^ lies in the height ratio of the first and second peak in the ρ_M^2+^_ profile, where Sr^2+^ strongly favors the 4D site, with around 80% of adsorbed Sr^2+^ occupying a 4D site. In contrast, Ba^2+^ does not have two separate peaks in the ρ_Ba^2+^_ profile and adsorbs almost exclusively at the 4D site (with minor adsorption on 2D and tridentate (3D) site), as shown in the inset of Figure 5a.

The NH-TiNP surface is an alternative representation of the TiNP/water interface, which has the same surface charge density as the H-TiNP surface; however, the concentration of surface hydroxyl groups bound to the terminal Ti atoms is much lower, resulting in strong adsorption of water molecules on the terminal Ti atoms (see Section 2.4.1 for details). This has a major influence on the adsorption of the ions. Figure 5b shows the ρ_M^2+^_ profiles for the ions adsorbed on the negatively charged NH-TiNP surface. Most striking are the much lower heights of the peaks in the density profiles compared with the H-TiNP model, indicating that adsorption is less likely for most ions in the case of the NH-TiNP surface, the only exception being Mg^2+^. Since Mg^2+^ is strongly hydrated, the effect of hydroxylation of the TiNP surface is not as obvious as for the other ions and even shows the opposite trend, where peak heights are increased for the NH-TiNP surface compared to its H-TiNP counterpart. Since there are many fewer hydroxyl groups on the NH-TiNP surface, both the water molecules and the ions can move closer to the last row of the crystal lattice of the TiNP surface (at *d* = 0 nm); hence, the ρ_M^2+^_ profiles for the NH-TiNP model are shifted slightly closer to *d* = 0 nm compared to the H-TiNP model.

For Ca^2+^ and Sr^2+^, the 4D and 2D peaks in ρ_M^2+^_ are less spatially separated, while the ions are adsorbing on the NH-TiNP surface rather than the H-TiNP one, suggesting that the adsorption becomes less specific when using the NH-TiNP model. While Ca^2+^ still adsorbs preferentially at the 2D site, the proportion of adsorption at the 4D and even the 3D (Figure S6 in the Supporting Information) site (which is almost absent in the H-TiNP model) is significantly increased (from around 10% with the H-TiNP model to around 40% with the NH-TiNP model), leading to a merging of the peaks in the density profile. The effect of hydroxylation is even more evident in the case of Sr^2+^, where adsorption on 2D sites becomes the main adsorption mode when most of the surface hydroxyl groups are removed. A similar trend is also observed for Ba^2+^, but here the 3D site becomes the most common. Since the NH-TiNP surface only has a small number of hydroxyl groups on the surface, most of the suitable 4D sites (usually consisting of two bridging oxygen atoms and two hydroxyl oxygen atoms) that are present with the use of the H-TiNP model are removed, and Ba^2+^ adsorbs in such a way that it is still coordinated by as many oxygen atoms of the TiNP surface as possible.

It should also be noted that on the NH-TiNP surface, some cations are adsorbed fully hydrated, as indicated by the peaks in the number density profiles starting around *d* = 0.5 nm (Figure 5b). For Mg^2+^, this is the main peak in the density profile, and for the other cations, the probability of adsorption at this position decreases with their size, which is recognizable by the lowering of the peak from Mg^2+^ to Ba^2+^. The peak position for fully hydrated adsorbed ions also depends on the size of the cation and moves farther from the TiNP surface with increasing ion size. Adsorption of fully hydrated ions is more likely in the case of the NH-TiNP surface as fewer cations are adsorbed directly on the surface, resulting in less electrostatic repulsion for the hydrated cations.

To estimate how favorable the adsorption of M^2+^ on the TiNP surface is energetically, PMF profiles of cations along the reaction coordinate perpendicular to the negatively charged H-TiNP surface were calculated by using umbrella sampling simulations. The H-TiNP surface was chosen for PMF calculations since the adsorption of cations on it was more specific and more favorable than on the NH-TiNP surface. The results are shown in Figure 7. PMF profile of Mg^2+^ again stands out against other cations, having a very large energy barrier around *d* ≈ 0.45 nm, which sits between two minima that belong to fully hydrated Mg^2+^ (*d* ≈ 0.55 nm) and Mg^2+^ adsorbed at the 2D site (*d* ≈ 0.38 nm). This barrier is the reason for the low adsorption density of Mg^2+^, as the more favorable binding positions (4D site at *d* ≈ 0.35 nm and the 2D site) are very unlikely to exist due to the very unfavorable dehydration of Mg^2+^. Ca^2+^ shows two minima near the TiNP surface that correspond to the first two peaks in the ρ_Ca^2+^_ profile. The 2D site is preferred by Ca^2+^ because the energy barrier for the 2D Ca^2+^ ion to reach the 4D site is high. This is due to Ca^2+^ being coordinated by 6 water molecules when adsorbed at the 2D site (Figure 8c) and only by 3 water molecules when moving to the 4D site (Figure 8b). The dehydration of Ca^2+^ seems to be so unfavorable that Ca^2+^ prefers to be adsorbed at the 2D site. The energy barrier is much lower for Sr^2+^, since Sr^2+^ can move from the 4D (Figure 8d) to the 2D site (Figure 8e) while retaining its coordinated water molecules, making the transition much more favorable. As expected, there is only one minimum in the PMF profile for Ba^2+^, which corresponds to the 4D adsorption site. The maximum depths of the energy minima are correlated with the molar mass of the cations, with Ba^2+^ being the most favorably adsorbed. Figure 8f also shows how well solvated Ba^2+^ remains when adsorbed at the 4D site, while the occupation of the 2D site by Ba^2+^ is almost absent in the simulations.

**Figure 7:**
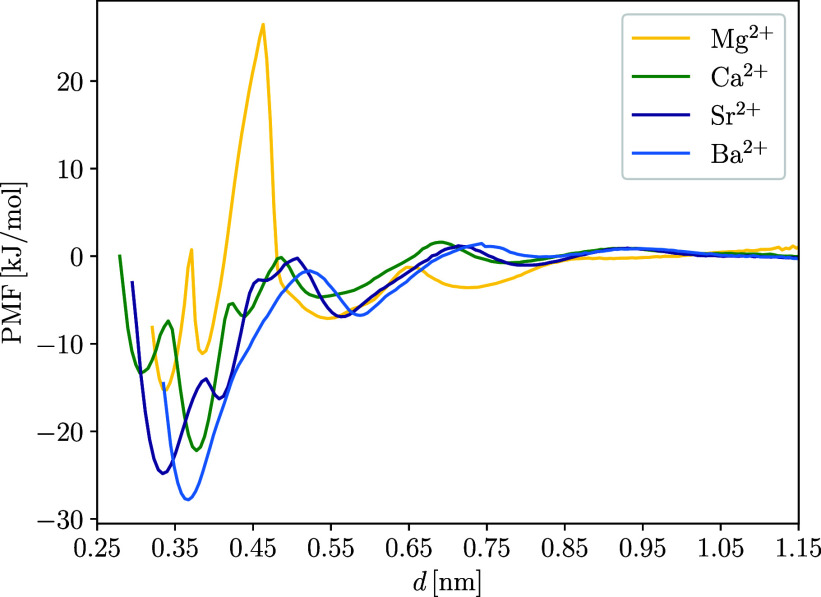
PMF profiles of M^2+^ during adsorption on the TiNP surface along a reaction coordinate perpendicular to the H-TiNP surface.

**Figure 8:**
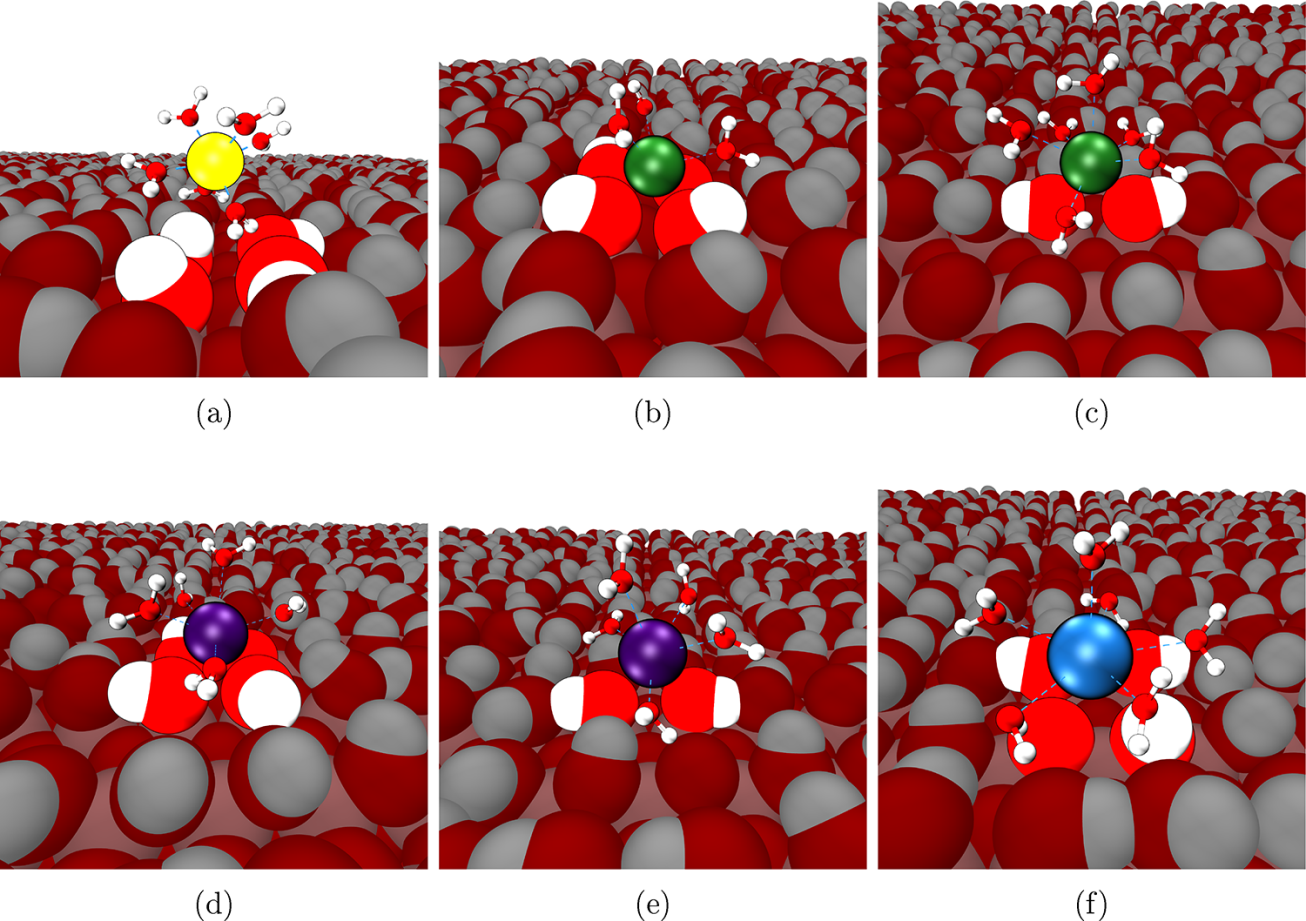
Typical geometries of adsorbed M^2+^, with the coordinated water molecules and the coordinated oxygen atoms of the TiNP surface highlighted (Mg^2+^ in yellow (a), Ca^2+^ in green (b, c), Sr^2+^ in purple (d, e), and Ba^2+^ in blue (f). Mg^2+^ (a) is fully hydrated and is not directly coordinated to any TiNP oxygen atoms. Ca^2+^ is adsorbed at the 4D (b) and 2D site (c), as is Sr^2+^ (4D in (d) and 2D in (e)). Ba^2+^ is shown adsorbed at the 4D site (f).

The difference between the value of PMF at *d* > 1 nm (where PMF reaches the value of around 0 kJ/mol) and the value of PMF at the position of the global minimum of the PMF profile can be used as an estimate of the free energy of adsorption (Δ*G*_ads,md_) of the cations on the TiNP surface. Fuerstenau and colleagues have calculated estimates of Δ*G*_ads,exp_ for M^2+^ on rutile from adsorption and electrokinetic measurements.^9^ The values of Δ*G*_ads,md_ obtained from our MD simulations are compared with their experimentally obtained values in Table 2.

**Table 2:** Values of Δ*G*_ads,md_ and Δ*G*_ads,exp_ for M^2+^ Adsorbed on the TiNP Surface^a^

	Δ*G*_ads,md_ [kJ/mol]	Δ*G*_ads,exp_ [kJ/mol]	Δ*G*_ads,md_/Δ*G*_ads,exp_ [%]
Mg^2+^	–15.26	–25.44	60
Ca^2+^	–22.20	–26.78	83
Sr^2+^	–24.82	–30.12	82
Ba^2+^	–27.82	–35.98	77

a Experimental values were obtained from the work of Fuerstenau et al.^9^

Comparison of the experimental and computer simulation values of Δ*G*_ads_ shows good qualitative agreement. The difference is largest in the case of Mg^2+^, where Δ*G*_ads,md_ is around 40% lower than the experimental value, which can still be regarded as relatively close when the simplicity of the MD model compared to the real system is taken into account. This result indicates that in real systems, the dehydration and adsorption of Mg^2+^ are somewhat more favorable than the MD model shows. However, for other M^2+^, the agreement is far better, with MD simulations understating the Δ*G*_ads,exp_ by about 20%, and, most importantly, the error in determination is very similar for Ca^2+^, Sr^2+^, and Ba^2+^, meaning that the MD model captures the differences between them very well.

### 3.4 Adsorption Affinity of M^2+^ and Their Effect on TiNP Surface Charge Calculated from Molecular Dynamics Simulations

To determine whether the MD model can capture the effects of metal ions on the zeta potential of TiNPs, we calculated the surface charge densities of TiNP surfaces with adsorbed ions (σ_TiNP+ions_). We define σ_TiNP+ions_ as

$$\sigma_{\mathrm{TiNP}+\mathrm{ions}}=\frac{2N_{\mathrm{ads}}(M^{2+})-N_{\mathrm{ads}}({\mathrm{Cl}}^{-})+\mathrm{TiNP}\;\mathrm{charge}}{\mathrm{surface}\;\mathrm{area}\;\mathrm{of}\;\mathrm{the}\;\mathrm{TiNP}\;\mathrm{slab}}$$ 1

where *N*_ads_(M^2+^) is the number of adsorbed M^2+^ and *N*_ads_(Cl^–^) is the number of adsorbed Cl^–^ (which was used as counterion in the simulations). The cutoff distance separating the adsorbed ions from the bulk solution was set at 1 nm and was determined from the density profiles of the cations and anions near the TiNP surface in a way that the main peaks in the density profiles were within the cutoff distance.

Although the zeta potential and σ_TiNP+ions_ are not directly comparable, both indicate how the total charge of TiNPs changes with the adsorption of ions and with the change in pH. For this reason, the *x*-axes in Figure 9 are reversed, as a positive TiNP surface charge density (σ_TiNP_) corresponds to a low pH and a negative σ_TiNP_ corresponds to a high pH. This allows an easier comparison with the zeta potential measurements in Figure 4.

**Figure 9:**
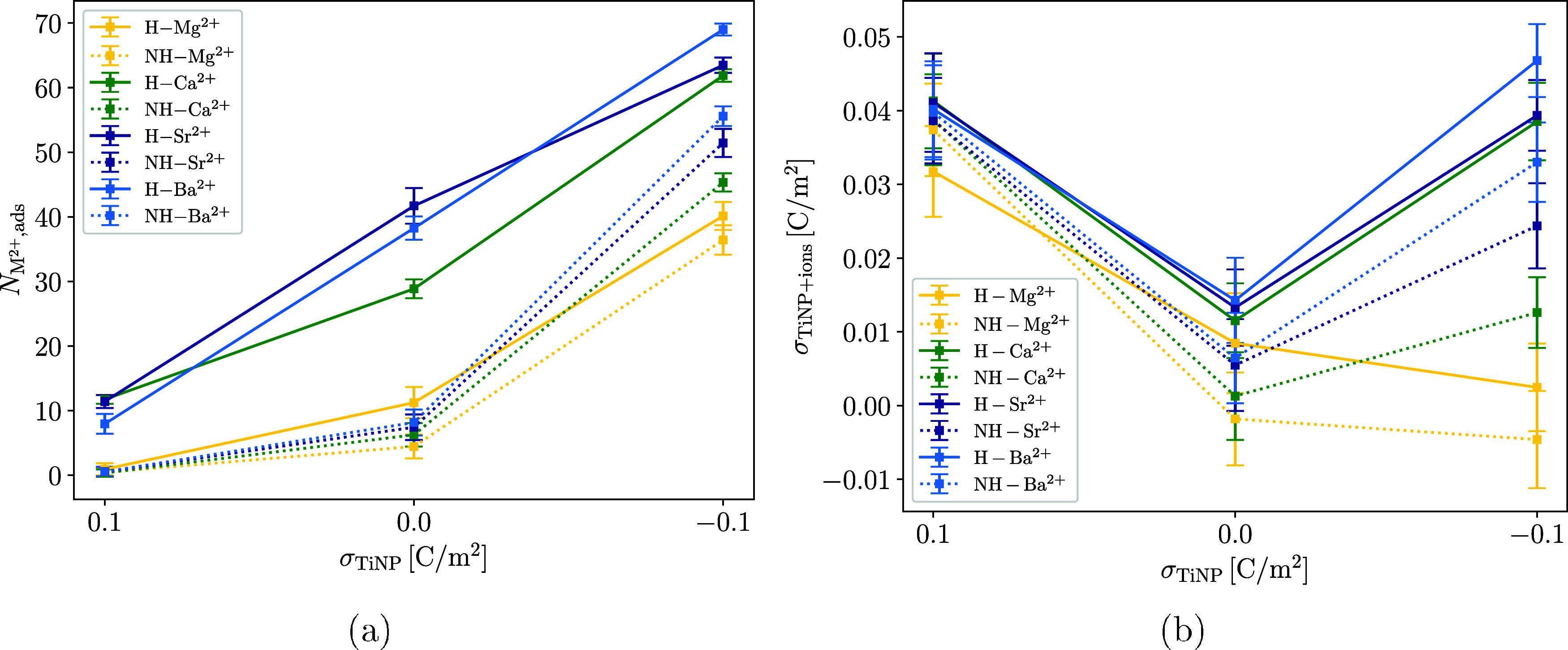
Dependence of the number of adsorbed M^2+^ ions (a) and the total charge density of the TiNP surface with adsorbed ions (σ_TiNP+ions_, b) on the surface charge density of the bare TiNP surface (σ_TiNP_), for the H-TiNP (H solid line) and the NH-TiNP (NH dotted line) surface. Note that the *x*-axes are reversed, as positive σ_TiNP_ corresponds to low pH and negative σ_TiNP_ corresponds to high pH. This allows an easier comparison with the zeta potential measurements in Figure 4. *c*(M^2+^) was about 200 mM in all simulations, which corresponds to 100 divalent cations in the simulation box.

Figure 9b shows the dependence of σ_TiNP+ions_ on σ_TiNP_ for the H-TiNP and NH-TiNP surfaces. The differences between the models are apparent mostly at σ_TiNP_ = −0.1 C/m^2^. Using the H-TiNP model, σ_TiNP+ions_ is less affected by the type of cation, except for Mg^2+^, where it continually decreases with decreasing σ_TiNP_. This trend is similar to the zeta potential trend in Figure 4d, where suspensions with added Ca^2+^, Sr^2+^, and Ba^2+^ all have a similar and relatively high zeta potential, while the zeta potential of suspensions with added Mg^2+^ is significantly lower.

For the NH-TiNP model, the trend of σ_TiNP+ions_ is quite different for all of the cations. It is more similar to the trend of the zeta potential in Figure 4c, where the zeta potential initially decreases close to 0 mV (near the isoelectric point of TiNPs) and then rises again as the pH is further increased but never reaches values as high as at the low pH. Both models of the TiNP surface capture the differences between the ions quite well, as both the amount of adsorbed cations and the σ_TiNP+ions_ roughly increase in the order: Mg^2+^ < Ca^2+^ < Sr^2+^ < Ba^2+^.

Even though the same concentrations of ions were used for simulations with both the H-TiNP and NH-TiNP surfaces, the adsorption of ions is much more favorable on the H-TiNP surface. In reality, the surface of TiNPs is most likely somewhere in between the H-TiNP and NH-TiNP models, with an equilibrium that shifts with the changing pH value of the suspension. At high pH, the surface is mostly hydroxylated, and the differences between cations start to become less noticeable (as in Figure 4c at pH > 9), while in the pH range where surface hydroxylation occurs only partially, the differences between the cations are more prominent (in Figure 4c for pH values between 6 and 8).

### 3.5 Competitive Adsorption between Metal Ions, Analyzed Using Molecular Dynamics Simulations

The MD simulations were performed similarly to the simulations in the previous sections, except that here a certain number of additional Na^+^ and a corresponding number of Cl^–^ were added to keep the simulation box electroneutral. The results for negatively charged H-TiNP (Figure 10) surfaces show that the addition of Na^+^ ions significantly decreases the amount of adsorbed M^2+^. The effect of Na^+^ decreases with the molar mass of M^2+^. Mechanistically, Na^+^ competes with M^2+^ for surface hydroxyl binding sites and screens the negative surface charge, thereby weakening the electrostatic driving force for divalent ion adsorption. The weaker binding of lighter, more strongly hydrated cations (e.g., Mg^2+^) makes them especially susceptible to replacement; indeed, nearly all Mg^2+^ is displaced when *c*_Na^+^_ > *c*_Mg^2+^_. For other divalent ions, the effect is less drastic since larger and more polarizable ions retain stronger adsorption via partial dehydration and inner-sphere complexation. The amount of adsorbed Ca^2+^ and Sr^2+^ is reduced by 57 and 49%, respectively, compared to solutions without sodium ions (when *c*_Na^+^_ is three times higher than *c*_M^2+^_). The adsorption of Ba^2+^ is least affected by the addition of Na^+^, with the number of adsorbed ions decreasing by around 40% under the same conditions as for Ca^2+^ and Sr^2+^. Comparison of XPS measurements (Figure 3) with MD simulations shows that the MD model severely overstates the removal of M^2+^ by Na^+^; however, the results are still in qualitative agreement. This is somewhat expected, as the MD model also understates the Δ*G*_ads_ values of divalent ions on TiNPs, indicating less favorable adsorption compared to real systems.

**Figure 10:**
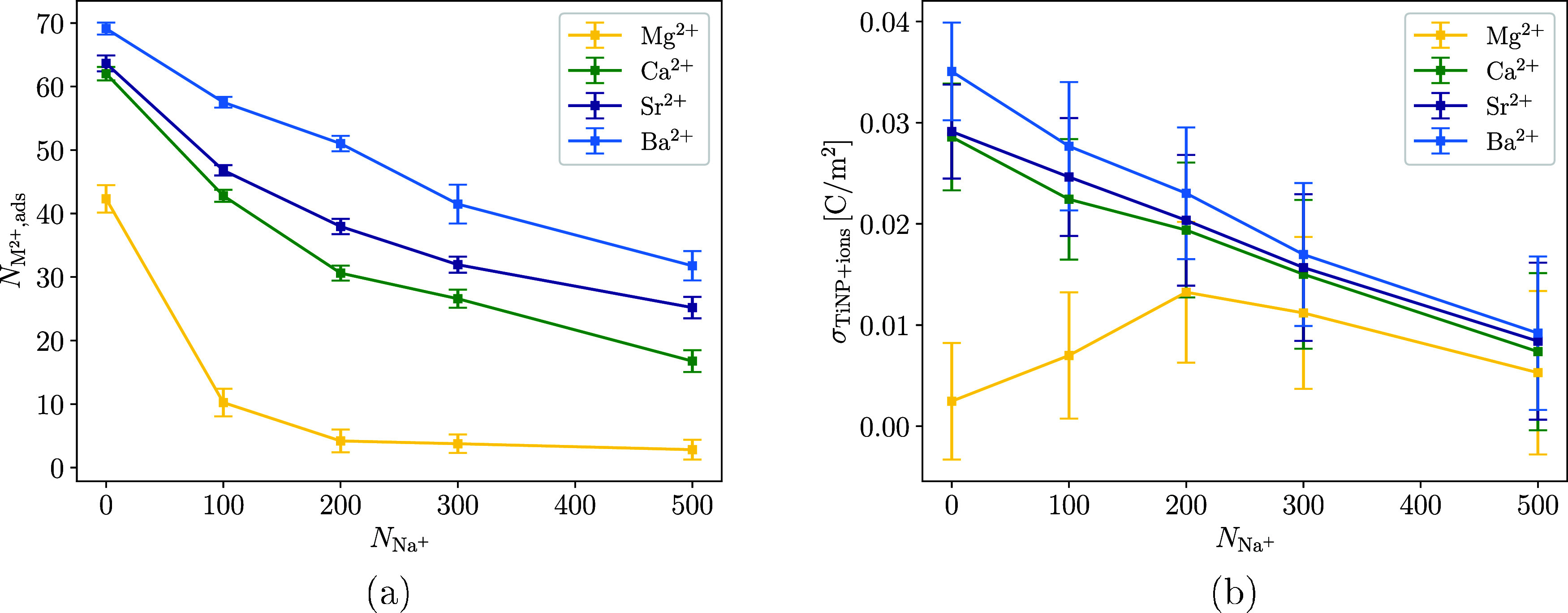
Number of adsorbed M^2+^ (*N*_M^2+^,ads_) on the H-TiNP surface as a function of the number of Na^+^ in (a) and the dependence of the total surface charge density of the H-TiNP surface with adsorbed ions (σ_TiNP+ions_) on the number of Na^+^ in the simulation box (b). *c*(M^2+^) was about 200 mM in all simulations, which corresponds to 100 divalent cations in the simulation box.

Figure 10b shows the influence of the Na^+^ concentration on the σ_TiNP+ions_. As the number of Na^+^ in the simulation box increases, σ_TiNP+ions_ decreases almost linearly for all M^2+^, with the exception of Mg^2+^, where it increases slightly. This leads to a reduction in the differences that divalent ions have on the surface charge of TiNP compared with TiNP suspensions without the addition of Na^+^. This is reasonable because, with lowered adsorption of M^2+^, TiNPs begin to behave like being in a suspension with a high concentration of monovalent salts, where the zeta potential starts to gravitate toward zero with increasing salt concentration.^59^

## 4 Conclusions

In this study, we investigated the interaction of M^2+^ cations with TiNPs using a combination of experimental techniques and MD simulations. Our experimental results, based on direct measurements using TEM and XPS, demonstrate that the adsorption affinity of divalent cations increases in the order: Mg^2+^ < Ca^2+^ ≈ Sr^2+^ < Ba^2+^. The impact of cation adsorption on the surface charge properties of TiNPs was further explored through zeta potential measurements across a broad concentration range (0.1–100 mM). These measurements are consistent with the adsorption data and show that the zeta potential shifts more significantly with heavier cations. At higher cation concentrations (100 mM), the differences in zeta potential between cations become less pronounced—except for Mg^2+^, which induces the least overcharging of TiNPs.

Our experimental findings were corroborated by extensive MD simulations. The simulations revealed that M^2+^ cations exhibit distinct adsorption preferences depending on the TiNP surface model. For the H-TiNP model, cation-specific differences were the most evident: Mg^2+^ remains fully hydrated with minimal specific adsorption, Ca^2+^ predominantly adsorbs in a 2D mode with limited affinity for 4D sites, Sr^2+^ adsorbs at both 2D and 4D sites with a preference for the latter, and Ba^2+^ adsorbs almost exclusively at the 4D sites. On the NH-TiNP surface, adsorption is generally less specific, though the same overall trend is maintained—heavier cations tend to coordinate with more surface oxygen atoms.

To relate these findings to surface charge properties, we computed the net surface charge of TiNPs with adsorbed cations from the MD simulations. The results closely align with the trends observed in zeta potential measurements, suggesting that real TiNP surfaces likely exhibit behavior intermediate between the H-TiNP and NH-TiNP models across the studied pH range.

The influence of monovalent cations on M^2+^ adsorption was also examined by using MD simulations. These results qualitatively match the XPS data, indicating that the presence of monovalent ions at concentrations exceeding those of divalent cations significantly reduces M^2+^ adsorption. This finding opens new avenues for studying ion-specific effects of monovalent cations in modulating divalent ion adsorption on TiNPs, as well as for exploring how the adsorption of different divalent ions—such as heavy metal ions—is influenced by the presence of monovalent ions in suspension.

This work builds upon previous experimental and theoretical studies of divalent ion adsorption on TiNPs, offering a more detailed understanding of the roles of ion identity, concentration, and competitive adsorption. Furthermore, this demonstrates that all investigated divalent cations can be effectively modeled using the selected TiNP surface models, providing a robust framework for studying more complex systems involving biological or synthetic molecules of interest.

## Supplementary Material



## Notes

XRD, TEM, and BET data for the TiNPs used in experiments, structures of H-TiNP and NH-TiNP surfaces used in MD simulations, examples of XPS spectra and Shirley background correction, model parameters used in MD simulations (partial charges, Lennard-Jones parameters, bond, and angle parameters), and 3D adsorption site structure on the NH-TiNP surface (PDF)
