# Supplementary material for: Role of Ion Size and Hydration in Competitive Adsorption of Alkaline Earth Metals on TiO2 Nanoparticles: Experimental and Molecular Dynamics Insights
Source: ACS Omega. 2025 Dec 15;10(51):62563–75. doi: 10.1021/acsomega.5c06067 (PMC12756764; doi:10.1021/acsomega.5c06067)
Supplement: Supplementary file 1 [file ao5c06067_si_001.pdf]

Role of Ion Size and Hydration in Competitive Adsorption of Alkaline Earth Metals on TiO_2 Nanoparticles: Experimental and Molecular Dynamics Insights

SUPPORTING INFORMATION

Tilen Berglez [a], Boštjan Genorio [a], Goran Dražić [b], Jurij Reščič [a,*], Klemen Bohinc [c*]

[a] Faculty of Chemistry and Chemical Technology, University of Ljubljana,
Večna pot 113, 1000 Ljubljana, Slovenia

[b] Department of Materials Chemistry, National Institute of Chemistry,
Hajdrihova ulica 19, 1000 Ljubljana, Slovenia

[c] Faculty of Health Sciences, University of Ljubljana,
Zdravstvena 5, 1000 Ljubljana, Slovenia

* Corresponding author, E-mail: jurij.rescic@fkkt.uni-lj.si, klemen.bohinc@zf.uni-lj.si

1 TiNP characterization

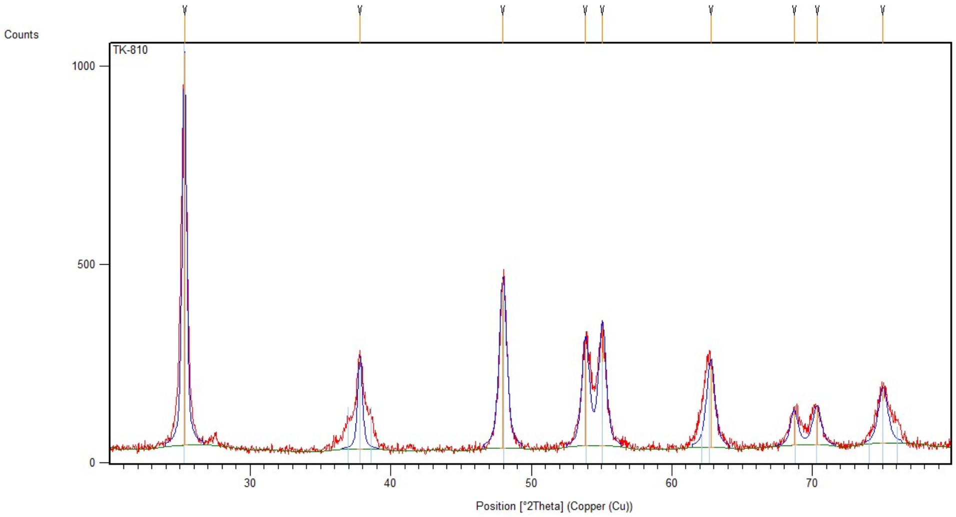

(a)

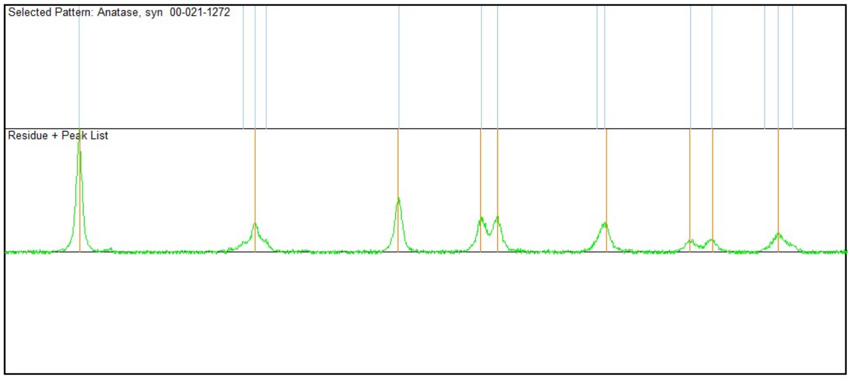

(b)

Figure S1: XRD spectrum of the TiNPs used in this work (a) and comparison of the peaks in the TiNP sample with the positions of peaks of pure anatase (b).

XRD measurements (Figure S1) of the purchased TiNPs show a very well defined anatase strutcture. Using the Scherrer relation, the primary crystalite size was estimated to be around 20 nm, which is in good agreement with the specified size of the TiNPs.

BET analysis showed that the specific surface of TiNPs was 91.0m^2/g.

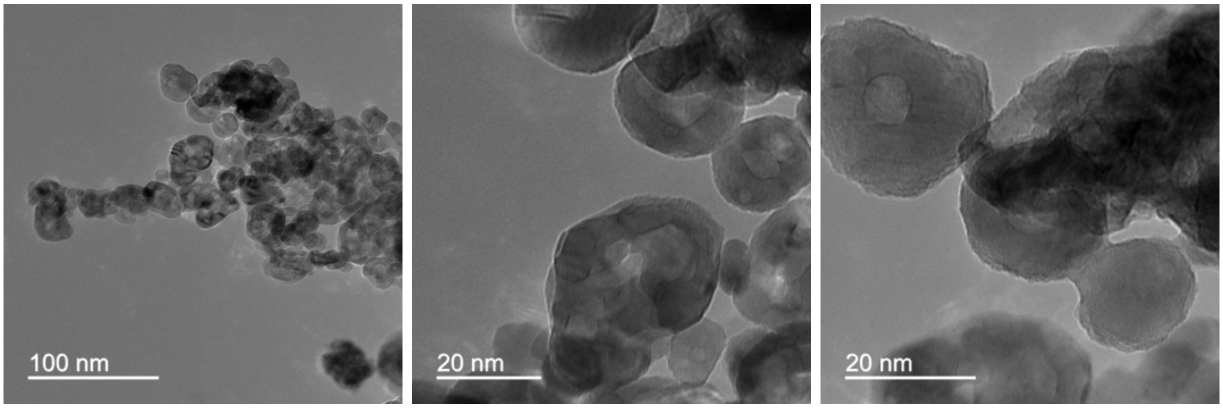

(a) (b) (c)

Figure S2: TEM micrographs of TiNPs used in this work at size scale of 100 nm (a), and at 20 nm at two different positions on the TEM grid (b,c). The size of primary particles is around 20-25 nm, very close to the size specified by the supplier (25 nm) and close to the crystalite size estimated from XRD measurements (20 nm).

2 TiNP model surface used in MD simulations

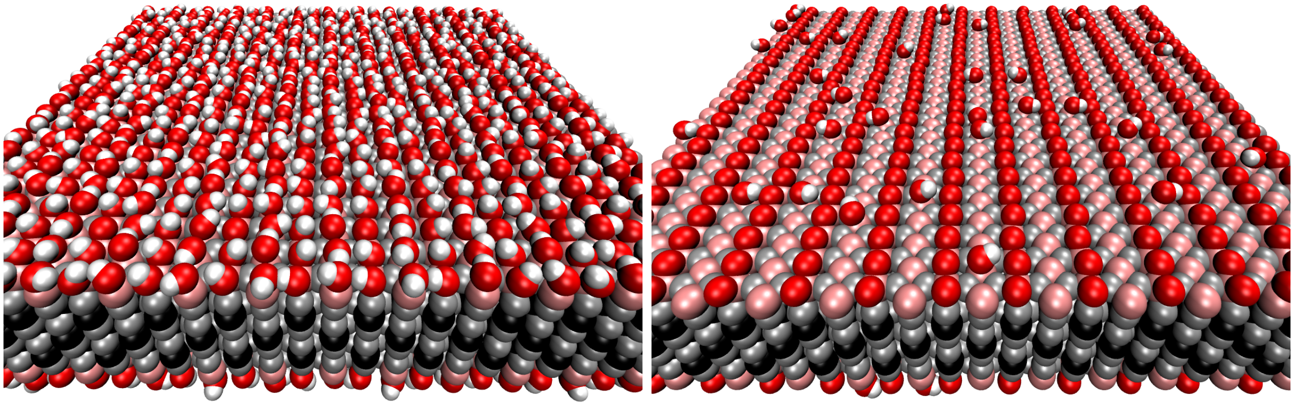

(a) (b)

Figure S3: Negatively charged hydroxylated (a) and non-hydroxylated (b) TiNP surface. Bulk Ti and O atoms are coloured black and silver, respectively, while the terminal Ti atoms are coloured light pink, the bridging and hydroxyl oxygen atoms are coloured red and the hydroxyl hydrogen atoms are coloured white.

3 XPS spectra of TiNPs with adsorbed cations

In this section, example of a XPS spectra is given for a sample which contained all of the investigated M^{2+} together with TiNPs. In Figure S4a the whole recorded spectrum is presented, while in the Figure S4b only the selected peaks that were used for quantification are showed together with the atomic % of elements in the sample (upper right corner of the figure).

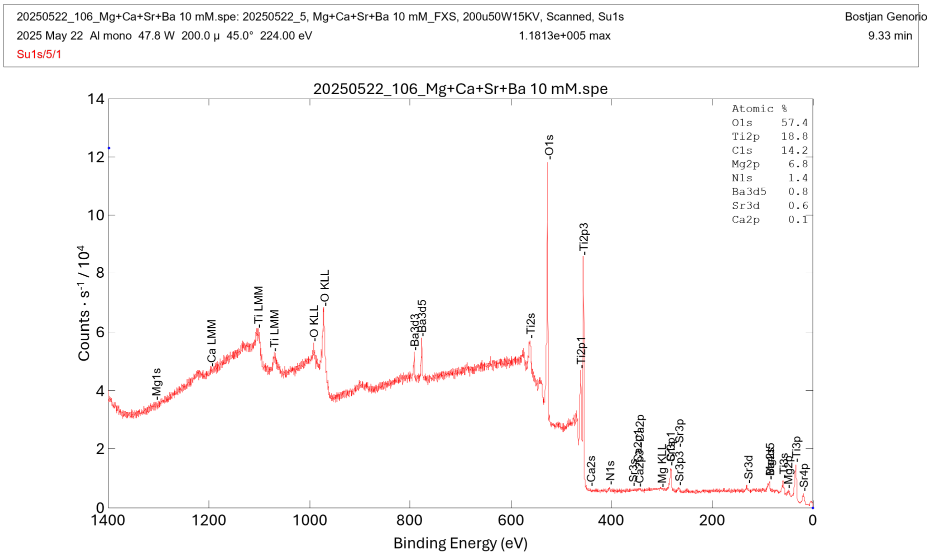

(a)

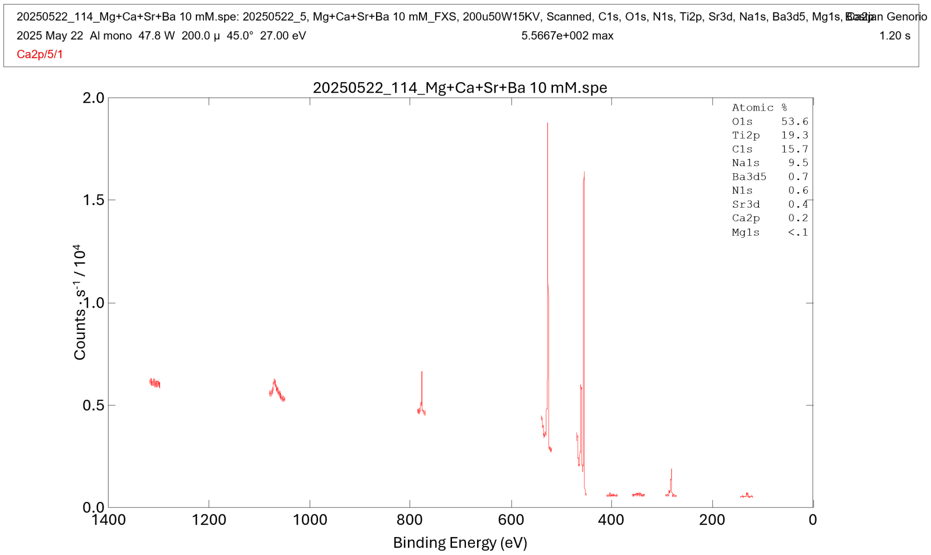

(b)

Figure S4: XPS spectrum of a TiNP sample collected from a suspension which contained all the investigated cations at a concentration of 10 mM (full spectrum (a) and selected peaks used for quantification (b)).

4 Examples of Shirley background correction

Examples of Shirley background correction for samples of TiNPs with added 10 mM $BaCl_2$ are shown in Figure S5.

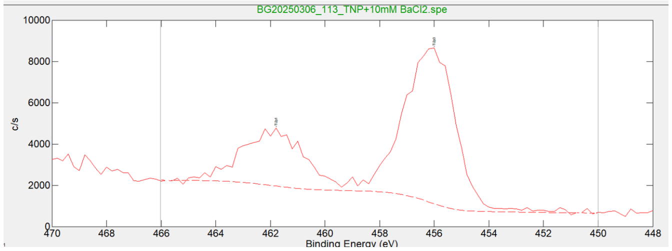

(a)

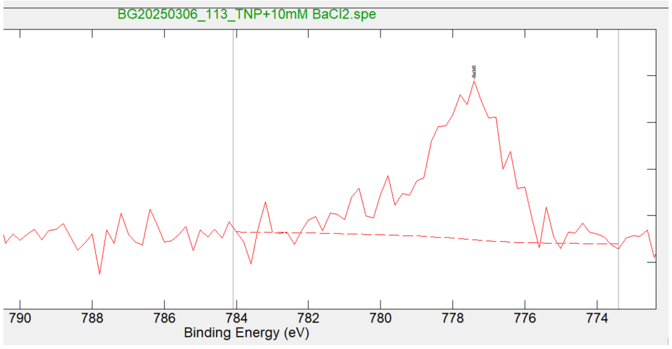

(b)

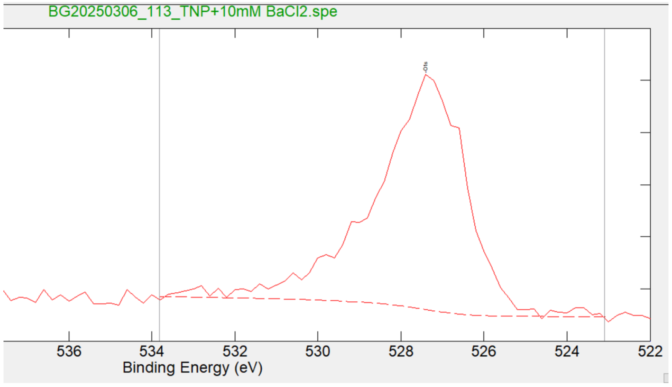

(c)

Figure S5: Shirley background correction on the XPS measurements of TiNPs with added 10 mM $BaCl_2$ for Ti 2p peaks (a), O 1s peak (b) and Ba 3d peak (c) that were used for quantification.

5 Model parameters for MD simulations

Table S1: Nonbonded parameters of atoms used in MD simulations. Monovalent cations had a charge of $0.75e$, anions $-0.75e$ and the divalent ions had a charge of $1.5e$ (when used in simulation with charged surfaces of TiNPs).

Atom type	σ (nm)	ϵ (kJ mol^{-1})
Ti	1.958×10^{-1}	2.5422
O	2.875×10^{-1}	1.3897
H	0.000×10^{0}	0.0000
Na	2.1150×10^{-1}	0.544284
Cl	4.10000×10^{-1}	0.492800
Mg	2.423×10^{-1}	0.0426867
Ca	2.913×10^{-1}	0.409531
Sr	3.197×10^{-1}	0.871377
Ba	3.528×10^{-1}	1.55337

Table S2: Partial charges (in e) for different TiNP models.

**Atom type**		**Charge (e)**
Bulk		
Bulk Ti	–	2.196
Bulk O	–	-1.098
Terminal Ti	–	2.162
Negative H-TiNP		
Hydroxylated Ti	–	2.151
Hydroxyl O	–	-0.944
Hydroxyl H	–	0.425
Bridging O	–	-0.970
Bridging H	–	0.450
Neutral H-TiNP		
Hydroxylated/Bridging Ti	–	2.196
Hydroxyl O	–	-1.008
Hydroxyl H	–	0.459
Bridging O	–	-1.035
Bridging H	–	0.486
Positive H-TiNP		
Hydroxylated/Bridging Ti	–	2.162
Hydroxyl O	–	-0.936
Hydroxyl H	–	0.434
Bridging O	–	-0.958
Bridging H	–	0.461
Negative NH-TiNP		
Hydroxylated/Bridging Ti	–	2.147
Hydroxyl O	–	-0.947
Hydroxyl H	–	0.421
Bridging O	–	-1.028
Positive NH-TiNP		
Hydroxylated/Bridging Ti	–	2.165
Bridging O	–	-1.007
Bridging H	–	0.465

Table S3: Bonded parameters used in the TiNP models. Bond lengths r_0 in nm, force constants k_b in kJ mol^{-1} nm^{-2}; angles θ_0 in degrees, force constants k_a in kJ mol^{-1} rad^{-2}.

**Bond**	r_0 (nm)	k_b (kJ mol^{-1} nm^{-2})
Ti–O	0.1895	845200.0
O–H	0.0983	463700.0
Angles		
**Angle**	θ_0 (deg)	k_a (kJ mol^{-1} rad^{-2})
Ti–O–H	90.85	59.15

6 3D adsorption site on the non-hydroxylated TiNP surface during MD simulations

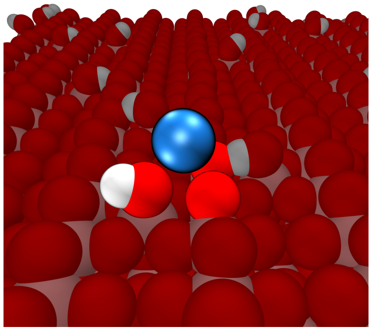

Figure S6: 3D adsorption site on the surface of the non-hydroxylated TiNP surface showing an adsorbed Ba^{2+} ion. Highlighted are the oxygen atoms of the TiNP surface to which the Ba^{2+} ion is directly coordinated
